# Iclaprim mesylate displaying a hydrogen-bonded mol­ecular tape

**DOI:** 10.1107/S2056989022011689

**Published:** 2023-01-01

**Authors:** Sandro Neuner, Thomas Gelbrich, Klaus Wurst, Josef Spreitz, Sven Nerdinger, Ulrich J. Griesser, Marijan Stefinovic, Herwig Schottenberger

**Affiliations:** a University of Innsbruck, Department of General, Inorganic and Theoretical Chemistry, Innrain 80-82, 6020 Innsbruck, Austria; b University of Innsbruck, Institute of Pharmacy, Innrain 52, 6020 Innsbruck, Austria; c Aglycon Dr. Spreitz KG, Europapark 1, A-8412 Allerheiligen b. Wildon, Austria; d Sandoz GmbH, Biochemiestrasse 10, 6250 Kundl, Austria; Vienna University of Technology, Austria

**Keywords:** crystal structure, hydrogen bonding, pharmaceuticals

## Abstract

The title compound, 2,6-di­amino-5-[(2-cyclo­propyl-7,8-dimeth­oxy-*2H*-1-benzo­pyran-5-yl)meth­yl]pyrimidin-1-ium methane­sulfonate, C_19_H_23_N_4_O_3_^+^·CH_3_O_3_S^−^, is a salt made up from a protonated iclaprim mol­ecule and a mesylate anion. The pyrimidine and chromene units of the iclaprim mol­ecule form an orthogonal arrangement [inter­planar angle of 89.67 (6)°], and the 3-nitro­gen position of the pyrimidine ring is protonated. Four distinct N—H⋯O inter­actions and an additional N—H⋯N hydrogen bond connect iclaprim and mesylate mol­ecules to one another, resulting in an infinite hydrogen-bonded mol­ecular tape structure. The central section of the tape is formed by a sequence of fused hydrogen-bonded rings involving four distinct ring types.

## Abstract 2

Iclaprim and mesylate mol­ecules are linked into a hydrogen-bonded mol­ecular tape, the central section of which is composed of fused rings.

## 1. Chemical context

Iclaprim is a di­hydro­folate reductase (DHFR) inhibiting anti­biotic containing a 2*H*-chromene structure that targets Gram-positive bacteria (Masciadri, 1997 ▸). The current study is part of an investigation aimed at improving the synthetic route to iclaprim and accessing its salts (Nerdinger *et al.*, 2020 ▸). 
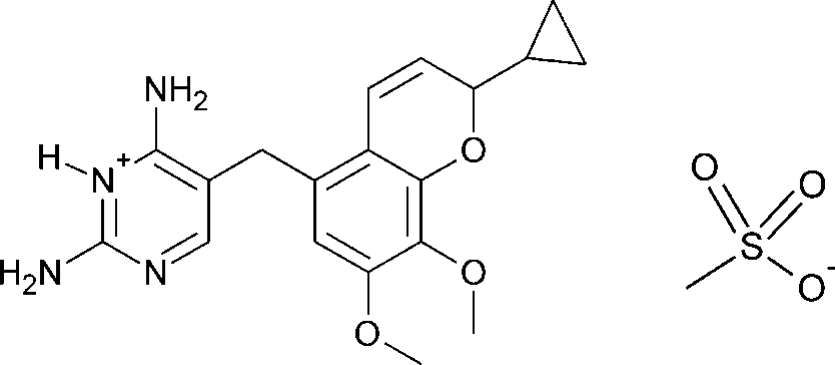


Iclaprim was synthesized according to the original route described by Jaeger *et al.* (2005 ▸), using 3-hy­droxy-4,5-di­meth­oxy­benzaldehyde (Cervi *et al.*, 2013 ▸), which was further purified by recrystallization from ethanol/*n*-hexane. We achieved a much better purity by trituration in hot ethanol and subsequent recrystallization from boiling aceto­nitrile. The title compound, (I), is the corresponding mesylate salt, and it was produced in a subsequent step.

## 2. Structural commentary

**Figure 1:**
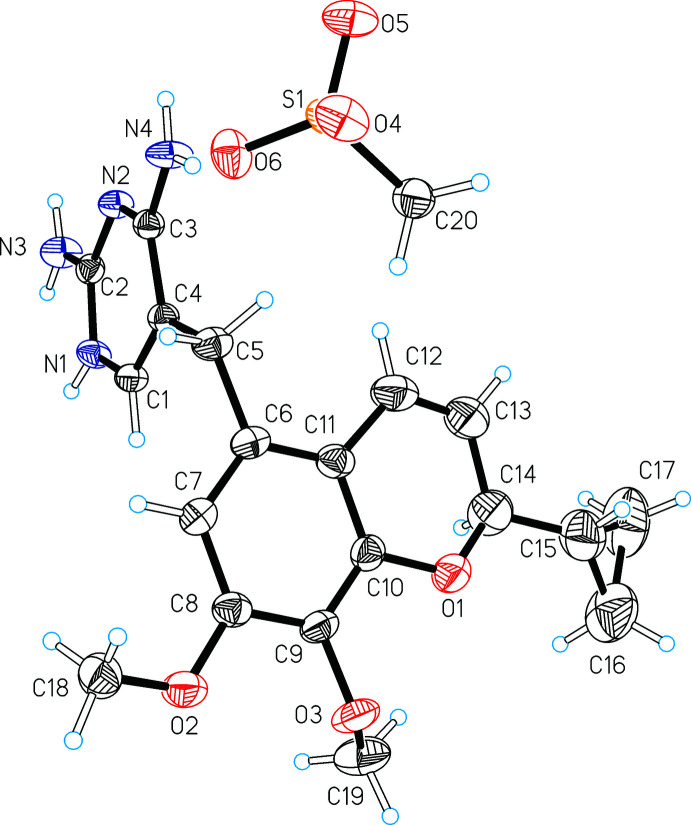
The structures of the mol­ecular entities with displacement ellipsoids drawn at the 50% probability level and hydrogen atoms drawn as spheres of arbitrary size.

The asymmetric unit of (I) consists of one formula unit, composed of an CH_3_SO_3_^−^ anion and an iclaprim cation in which the 3-nitro­gen atom of the pyrimidine ring is protonated, *i.e.* N1 (Fig. 1 ▸). The mol­ecular conformation of the iclaprim mol­ecule is largely defined by the relative arrangement of the essentially planar pyrimidine and chromene units. The CH_2_ carbon atom C5 links the pyrimidine ring (C1, N1, C2, N2, C3, C4) with the fused benzene ring of the chromene unit (C6, C7, C8, C9, C10, C11). With regard to the two bridging bonds, the torsion angles C3—C4—C5—C6 [–160.8 (2)°] and C4—C5—C6—C7 [–96.5 (3)°] indicate that the C5—C6 bond is twisted slightly out of the pyrimidine plane, whilst the C4—C5 bond is oriented approximately perpendicular to the benzene ring. Accordingly, the two six-membered rings linked *via* C5 form an orthogonal arrangement with an inter­planar angle of 89.67 (6)°. In the chromene moiety, the 7-meth­oxy substituent is significantly twisted out of the ring plane [C10—C9—O3—C19 = −70.3 (3)°], whilst the 8-meth­oxy substituent is almost coplanar with the plane of the fused benzene ring [C9—C8—O2—C18 = 167.6 (2)°]. The 2*H*-pyran ring displays the expected bond lengths [C12—C13 = 1.323 (4) Å]. The program *PLATON* (Spek, 2020 ▸) was used to calculate puckering parameters (Cremer & Pople, 1975 ▸) for the 2*H*-pyran ring. The obtained values, *θ* = 65.5 (7)°, *φ* = 328.4 (7)° and *q* = 0.253 (3) Å, are consistent with the presence of a skew-boat conformation (Boeyens, 1978 ▸).

## 3. Supra­molecular features

**Figure 2:**
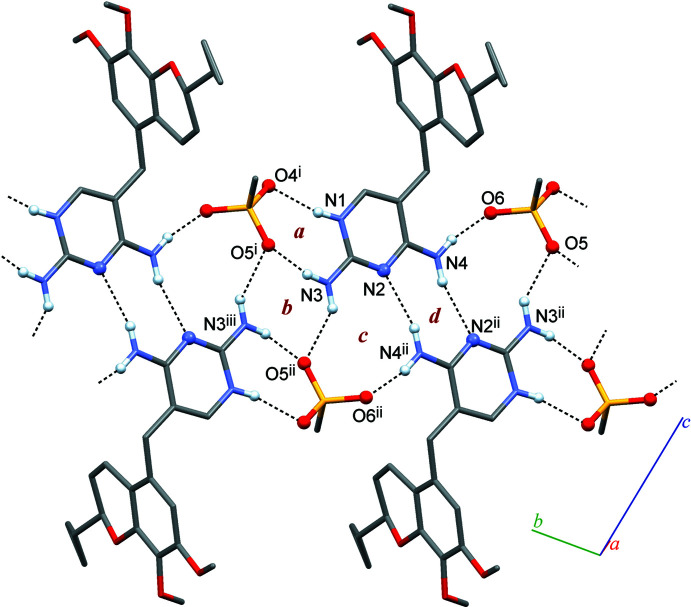
Tape structure composed of N—H⋯O and N—H⋯N-bonded iclaprim and mesylate mol­ecules, based on four essential ring motifs (*a*–*d*). [Symmetry codes: (i) *x* − 1, *y* + 1, *z*; (ii) −*x*, −*y* + 1, −*z*; (iii) −*x* − 1, *y* + 2, *z*.]

The iclaprim mol­ecule displays two NH_2_ groups attached to the pyrimidine ring (N3, N4) and the protonated N1 atom of the pyrimidine ring as potential hydrogen-bond donor groups. These hydrogen-bond donor functions are engaged in five distinct inter­molecular N—H⋯*A* inter­actions (Table 1 ▸). N1 and N3 are linked to two O sites, each belonging to the same mesylate anion, *i.e.* N1—H1*N*⋯O4^i^ and N3—H3*A*⋯O5^i^. In Fig. 2 ▸, the resulting ring motif is denoted as *a*, and it has the graph-set symbol 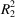 (8) (Etter *et al.*, 1990 ▸; Bernstein *et al.*, 1995 ▸). N3 is additionally linked, *via* an N3—H3*B*⋯O5^ii^ inter­action, to a second mesylate unit. The resulting centrosymmetric ring *b* (Fig. 2 ▸) comprises two iclaprim and two mesylate units (with O5 accepting two hydrogen bonds) and is described by the symbol 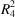 (8). The second NH_2_ group forms an N4—H4*B*⋯O6 inter­action with a mesylate anion, and it is also hydrogen-bonded to the unprotonated pyrimidine N atom of a second iclaprim mol­ecule *via* N4—H4*A*⋯N2^ii^. The latter two inter­actions generate two additional ring motifs, namely the 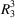 (10) ring *c* linking two pyrimidine mol­ecules with one anion and the centrosymmetric 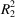 (8) ring *d*. The diagram in Fig. 2 ▸ illustrates that certain hydrogen-bonded rings are fused together because of shared N—H⋯*A* inter­actions, *i.e. a* + *b*, *b* + *c* and *c* + *d*. Altogether, the five distinct inter­actions listed in Table 1 ▸ result in a one-dimensional extended mol­ecular tape structure of hydrogen-bonded iclaprim and mesylate units propagating parallel to [ 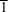 10]. The iclaprim mol­ecule is bonded to two different mesylate anions, one is a two-point and the other a one-point connection. It is also two-point connected to a neighbouring iclaprim mol­ecule. In turn, the mesylate anion accepts four hydrogen-bonds from three iclaprim mol­ecules, and all of its O atoms participate in hydrogen bonding.

## 4. Database survey

The Cambridge Structural Database (version 5.43, September 2022; Groom *et al.*, 2016 ▸) contains two other examples of mol­ecules displaying the 7,8-dimeth­oxy-2*H*-chromene fragment, namely methyl­ripariochromene A (Guerin *et al.*, 1989 ▸; CSD refcode JAZLIF) and 6,7,8-tri­meth­oxy­coumarin (Saidi *et al.*, 2007 ▸; CSD refcode KIKDOY). In each case, the 7- and 8-meth­oxy substituents are significantly twisted out of the ring plane as shown by the corresponding torsion angles, *i.e.* mol­ecule *A* of JAZLIF: 63.4°,–66.2°; mol­ecule *B* of JAZLIF: −140.2°, 89.4°; KIKDOY: 88.0, −110.9°.

## 5. Synthesis and crystallization

**Figure 3:**
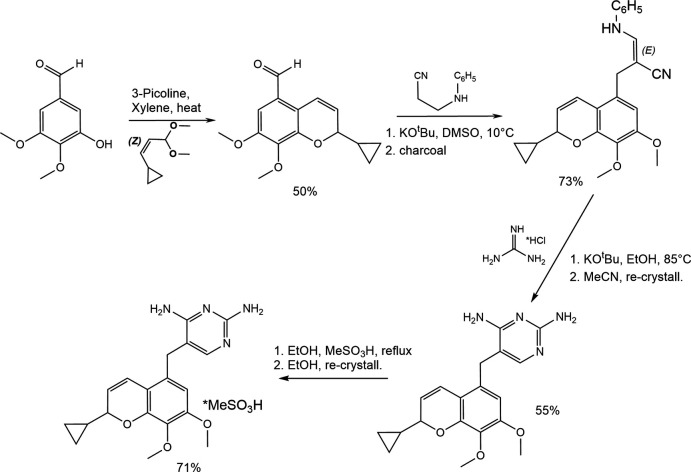
Synthesis scheme to prepare iclaprim.

Iclaprim mesylate was prepared according to a modified procedure based on the original synthesis by Jaeger *et al.* (2005 ▸) shown in Fig. 3 ▸. The iclaprim free base (500 mg, 1.41 mmol) was suspended in 75 ml of aceto­nitrile and heated to reflux. The resulting clear solution was slowly cooled to room temperature overnight and then kept at 253 K to complete the crystallization process. The resulting white solid was isolated by filtration and dried under high vacuum at room temperature. The obtained iclaprim free base (1.00 g, 2.82 mmol) was recrystallized in aceto­nitrile and was suspended in 35 ml of ethanol and heated to reflux. Heating was inter­rupted and a solution of 183 ml methyl­sulfonic acid (2.82 mmol) in 5 ml of ethanol was added in a dropwise manner. Refluxing was resumed and a further 10 ml of ethanol were added to obtain a clear solution. The solution was concentrated and allowed to cool slowly to room temperature, at which point aggregates of colourless columnar crystals started to form. The crystals were isolated *via* filtration and dried under high vacuum overnight; yield: 900 mg (71%).

## 6. Refinement

Crystal data, data collection and structure refinement details are summarized in Table 2 ▸. The structure was refined as a two-component twin with the components being related by a 179.9° rotation about the *a* axis. The refined value of the minor twin component fraction was 0.260 (1). All H atoms were identified in difference-Fourier maps and those of NH and NH_2_ groups were refined with a restrained N—H distance of 0.88 (2) Å and their *U*_iso_ parameters refined freely. The H atoms at the cyclo­propyl ring (C15, C16, C17) were refined with a restrained C—H distance of 0.96 (2) Å and with *U*_iso_(H) = 1.2*U*_eq_(C). Other H atoms bonded to secondary CH_2_ (C—H = 0.98 Å) or aromatic CH (C—H = 0.94 Å) carbon atoms were positioned geometrically. Their *U*_iso_ parameters were set to 1.2*U*_eq_(C). Methyl H atoms were idealized and included as rigid groups allowed to rotate but not tip (C—H = 0.97 Å) and their *U*_iso_ parameters were set to 1.5 *U*_eq_(C) of the parent carbon atom.

## Supplementary Material

Crystal structure: contains datablock(s) I. DOI: 10.1107/S2056989022011689/wm5666sup1.cif

Structure factors: contains datablock(s) I. DOI: 10.1107/S2056989022011689/wm5666Isup2.hkl

Supporting information file. DOI: 10.1107/S2056989022011689/wm5666Isup3.cml

CCDC reference: 2224639

Additional supporting information:  crystallographic information; 3D view; checkCIF report

## Figures and Tables

**Table 1:** Hydrogen-bond geometry (Å, °)

*D*—H⋯*A*	*D*—H	H⋯*A*	*D*⋯*A*	*D*—H⋯*A*
N1—H1*N*⋯O4^i^	0.85 (3)	1.99 (3)	2.827 (2)	168 (3)
N3—H3*A*⋯O5^i^	0.86 (2)	2.06 (2)	2.896 (3)	166 (2)
N3—H3*B*⋯O5^ii^	0.87 (2)	2.15 (2)	2.871 (3)	140 (2)
N4—H4*A*⋯N2^ii^	0.87 (2)	2.27 (2)	3.107 (3)	163 (2)
N4—H4*B*⋯O6	0.86 (2)	2.11 (2)	2.948 (3)	163 (2)

Symmetry codes: (i) 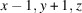 ; (ii) 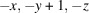 .

**Table 2:** Experimental details

Crystal data	
Chemical formula	C_19_H_23_N_4_O_3_^+^·CH_3_O_3_S^−^
*M*_r_	450.51
Crystal system, space group	Triclinic, *P* 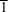
Temperature (K)	223
*a*, *b*, *c* (Å)	5.4726 (3), 8.8450 (4), 22.1395 (11)
α, β, γ (°)	98.094 (2), 93.754 (2), 98.919 (2)
*V* (Å^3^)	1043.98 (9)
*Z*	2
Radiation type	Mo *K*α
μ (mm^−1^)	0.20
Crystal size (mm)	0.21 × 0.18 × 0.03

Data collection	
Diffractometer	Bruker D8 QUEST PHOTON 100
Absorption correction	Multi-scan (*TWINABS*; Bruker, 2013 ▸)
*T*_min_, *T*_max_	0.910, 0.971
No. of measured, independent and observed [*I* > 2σ(*I*)] reflections	3851, 3851, 3507
(sin θ/λ)_max_ (Å^−1^)	0.604

Refinement	
*R*[*F* ^2^ > 2σ(*F* ^2^)], *wR*(*F* ^2^), *S*	0.043, 0.104, 1.08
No. of reflections	3851
No. of parameters	320
No. of restraints	10
H-atom treatment	H atoms treated by a mixture of independent and constrained refinement
Δρ_max_, Δρ_min_ (e Å^−3^)	0.57, −0.31

Computer programs: *APEX3* and *SAINT* (Bruker, 2013 ▸), *SHELXT* (Sheldrick, 2015*a* ▸), *SHELXL* (Sheldrick, 2015*b* ▸), *XP* in *SHELXTL* (Sheldrick, 2008 ▸), *Mercury* (Macrae *et al.*, 2020 ▸), *PLATON* (Spek, 2020 ▸) and *publCIF* (Westrip, 2010 ▸).

## supplementary crystallographic information

### Crystal data

**Table:**

C_19_H_23_N_4_O_3_^+^·CH_3_O_3_S^−^	*Z* = 2
*M**_r_* = 450.51	*F*(000) = 476
Triclinic, *P*1	*D*_x_ = 1.433 Mg m^−^^3^
*a* = 5.4726 (3) Å	Mo *K*α radiation, λ = 0.71073 Å
*b* = 8.8450 (4) Å	Cell parameters from 9886 reflections
*c* = 22.1395 (11) Å	θ = 2.3–25.3°
α = 98.094 (2)°	µ = 0.20 mm^−^^1^
β = 93.754 (2)°	*T* = 223 K
γ = 98.919 (2)°	Prism, colourless
*V* = 1043.98 (9) Å^3^	0.21 × 0.18 × 0.03 mm

### Data collection

**Table:**

Bruker D8 QUEST PHOTON 100 diffractometer	3851 measured reflections
Radiation source: Incoatec Microfocus	3851 independent reflections
Multi layered optics monochromator	3507 reflections with *I* > 2σ(*I*)
Detector resolution: 10.4 pixels mm^-1^	θ_max_ = 25.4°, θ_min_ = 2.4°
φ and ω scans	*h* = −6→6
Absorption correction: multi-scan (*TWINABS*; Bruker, 2013)	*k* = −10→10
*T*_min_ = 0.910, *T*_max_ = 0.971	*l* = −26→10

### Refinement

**Table:**

Refinement on *F*^2^	Secondary atom site location: difference Fourier map
Least-squares matrix: full	Hydrogen site location: mixed
*R*[*F*^2^ > 2σ(*F*^2^)] = 0.043	H atoms treated by a mixture of independent and constrained refinement
*wR*(*F*^2^) = 0.104	*w* = 1/[σ^2^(*F*_o_^2^) + (0.0406*P*)^2^ + 0.7995*P*] where *P* = (*F*_o_^2^ + 2*F*_c_^2^)/3
*S* = 1.08	(Δ/σ)_max_ = 0.001
3851 reflections	Δρ_max_ = 0.57 e Å^−^^3^
320 parameters	Δρ_min_ = −0.30 e Å^−^^3^
10 restraints	Extinction correction: SHELXL, Fc^*^=kFc[1+0.001xFc^2^λ^3^/sin(2θ)]^-1/4^
Primary atom site location: structure-invariant direct methods	Extinction coefficient: 0.026 (3)

### Special details

**Table:**

Geometry. All esds (except the esd in the dihedral angle between two l.s. planes) are estimated using the full covariance matrix. The cell esds are taken into account individually in the estimation of esds in distances, angles and torsion angles; correlations between esds in cell parameters are only used when they are defined by crystal symmetry. An approximate (isotropic) treatment of cell esds is used for estimating esds involving l.s. planes.

### Fractional atomic coordinates and isotropic or equivalent isotropic displacement parameters (Å^2^)

**Table:**

	*x*	*y*	*z*	*U*_iso_*/*U*_eq_	
O1	0.3058 (4)	0.7951 (2)	0.38547 (8)	0.0428 (5)	
O2	0.9479 (4)	1.1480 (2)	0.33158 (8)	0.0413 (5)	
O3	0.6716 (4)	1.0418 (2)	0.41560 (7)	0.0418 (5)	
N1	0.0557 (4)	0.9523 (2)	0.10508 (8)	0.0239 (4)	
H1N	0.015 (5)	1.042 (3)	0.1116 (12)	0.035 (7)*	
N2	0.0189 (3)	0.7157 (2)	0.03914 (8)	0.0240 (4)	
N3	−0.2450 (4)	0.8895 (2)	0.02407 (10)	0.0314 (5)	
H3A	−0.292 (5)	0.976 (2)	0.0359 (11)	0.030 (7)*	
H3B	−0.318 (5)	0.831 (3)	−0.0091 (10)	0.041 (8)*	
N4	0.2945 (4)	0.5524 (2)	0.05307 (9)	0.0296 (4)	
H4A	0.215 (5)	0.491 (3)	0.0214 (10)	0.042 (8)*	
H4B	0.416 (4)	0.528 (3)	0.0742 (11)	0.034 (7)*	
C1	0.2336 (4)	0.9125 (2)	0.14129 (10)	0.0230 (5)	
H1	0.3024	0.9821	0.1765	0.028*	
C2	−0.0565 (4)	0.8502 (2)	0.05556 (9)	0.0219 (4)	
C3	0.2094 (4)	0.6806 (2)	0.07298 (9)	0.0216 (4)	
C4	0.3182 (4)	0.7772 (2)	0.12931 (9)	0.0210 (4)	
C5	0.5138 (5)	0.7290 (3)	0.17050 (10)	0.0287 (5)	
H5A	0.4704	0.6177	0.1715	0.034*	
H5B	0.6731	0.7464	0.1527	0.034*	
C6	0.5457 (4)	0.8135 (3)	0.23546 (10)	0.0263 (5)	
C7	0.7329 (4)	0.9416 (3)	0.25216 (10)	0.0280 (5)	
H7	0.8359	0.9748	0.2226	0.034*	
C8	0.7705 (4)	1.0212 (3)	0.31165 (10)	0.0291 (5)	
C9	0.6184 (5)	0.9716 (3)	0.35590 (10)	0.0292 (5)	
C10	0.4339 (5)	0.8453 (3)	0.33923 (10)	0.0289 (5)	
C11	0.3909 (5)	0.7643 (3)	0.27896 (10)	0.0297 (5)	
C12	0.1991 (6)	0.6280 (3)	0.26835 (13)	0.0480 (7)	
H12	0.1796	0.5622	0.2305	0.058*	
C13	0.0514 (6)	0.5956 (3)	0.31140 (14)	0.0506 (8)	
H13	−0.0684	0.5052	0.3038	0.061*	
C14	0.0691 (5)	0.6975 (4)	0.37117 (13)	0.0477 (7)	
H14	−0.0506	0.7688	0.3658	0.057*	
C15	0.0006 (7)	0.6284 (4)	0.42415 (16)	0.0603 (9)	
H15	0.088 (6)	0.537 (3)	0.4274 (16)	0.072*	
C16	−0.0784 (9)	0.7122 (6)	0.47930 (19)	0.0775 (11)	
H16A	−0.051 (8)	0.687 (5)	0.5200 (11)	0.093*	
H16B	−0.082 (9)	0.821 (3)	0.4736 (19)	0.093*	
C17	−0.2553 (8)	0.5873 (6)	0.4384 (2)	0.0800 (12)	
H17A	−0.310 (8)	0.490 (3)	0.4518 (19)	0.096*	
H17B	−0.369 (7)	0.614 (5)	0.4075 (15)	0.096*	
C18	1.1370 (5)	1.1840 (3)	0.29246 (12)	0.0407 (6)	
H18A	1.2567	1.2718	0.3128	0.061*	
H18B	1.0637	1.2093	0.2549	0.061*	
H18C	1.2199	1.0953	0.2830	0.061*	
C19	0.4888 (7)	1.1252 (4)	0.44003 (14)	0.0568 (8)	
H19A	0.5389	1.1668	0.4827	0.085*	
H19B	0.3308	1.0562	0.4368	0.085*	
H19C	0.4719	1.2095	0.4172	0.085*	
S1	0.73446 (11)	0.29318 (6)	0.12358 (2)	0.02483 (16)	
O4	0.9826 (3)	0.26069 (18)	0.14030 (8)	0.0348 (4)	
O5	0.5834 (3)	0.16098 (19)	0.08426 (8)	0.0381 (4)	
O6	0.7365 (3)	0.43739 (19)	0.09992 (8)	0.0362 (4)	
C20	0.5874 (5)	0.3152 (3)	0.19206 (11)	0.0339 (6)	
H20A	0.5974	0.2263	0.2127	0.051*	
H20B	0.4145	0.3227	0.1827	0.051*	
H20C	0.6694	0.4086	0.2185	0.051*	

### Atomic displacement parameters (Å^2^)

**Table:**

	*U*^11^	*U*^22^	*U*^33^	*U*^12^	*U*^13^	*U*^23^
O1	0.0498 (11)	0.0446 (10)	0.0295 (9)	−0.0066 (9)	0.0072 (8)	0.0039 (8)
O2	0.0414 (11)	0.0421 (10)	0.0327 (9)	−0.0078 (8)	0.0035 (8)	−0.0043 (8)
O3	0.0470 (12)	0.0497 (11)	0.0230 (8)	0.0026 (9)	0.0004 (8)	−0.0070 (7)
N1	0.0272 (10)	0.0169 (9)	0.0275 (10)	0.0082 (8)	−0.0012 (8)	0.0003 (7)
N2	0.0252 (10)	0.0222 (9)	0.0236 (9)	0.0068 (8)	−0.0005 (8)	−0.0014 (7)
N3	0.0311 (11)	0.0299 (11)	0.0323 (11)	0.0133 (9)	−0.0084 (9)	−0.0021 (9)
N4	0.0301 (11)	0.0273 (10)	0.0288 (10)	0.0134 (9)	−0.0077 (9)	−0.0094 (8)
C1	0.0248 (11)	0.0207 (10)	0.0221 (10)	0.0037 (9)	−0.0009 (9)	−0.0002 (8)
C2	0.0227 (11)	0.0224 (10)	0.0214 (10)	0.0048 (9)	0.0037 (9)	0.0041 (8)
C3	0.0221 (11)	0.0206 (10)	0.0217 (10)	0.0036 (9)	0.0026 (9)	0.0018 (8)
C4	0.0222 (11)	0.0204 (10)	0.0201 (10)	0.0041 (9)	0.0025 (9)	0.0013 (8)
C5	0.0334 (13)	0.0278 (11)	0.0256 (11)	0.0133 (10)	−0.0025 (10)	−0.0002 (9)
C6	0.0306 (12)	0.0270 (11)	0.0226 (11)	0.0145 (10)	−0.0033 (9)	0.0003 (9)
C7	0.0283 (12)	0.0320 (12)	0.0247 (11)	0.0085 (10)	0.0025 (9)	0.0040 (9)
C8	0.0291 (13)	0.0280 (11)	0.0288 (12)	0.0052 (10)	−0.0027 (10)	0.0017 (9)
C9	0.0342 (13)	0.0318 (12)	0.0205 (11)	0.0071 (10)	−0.0019 (9)	0.0005 (9)
C10	0.0348 (13)	0.0284 (11)	0.0251 (11)	0.0090 (10)	0.0017 (10)	0.0059 (9)
C11	0.0321 (13)	0.0267 (11)	0.0291 (11)	0.0064 (10)	−0.0038 (11)	0.0020 (9)
C12	0.0526 (18)	0.0426 (15)	0.0401 (15)	−0.0066 (14)	0.0002 (14)	−0.0057 (12)
C13	0.0493 (18)	0.0459 (16)	0.0488 (17)	−0.0112 (14)	0.0013 (14)	0.0034 (13)
C14	0.0366 (15)	0.0582 (18)	0.0450 (16)	−0.0016 (13)	0.0020 (13)	0.0079 (13)
C15	0.061 (2)	0.0572 (19)	0.062 (2)	−0.0019 (17)	0.0203 (17)	0.0109 (16)
C16	0.072 (3)	0.097 (3)	0.064 (2)	0.002 (3)	0.026 (2)	0.019 (2)
C17	0.046 (2)	0.112 (3)	0.089 (3)	0.002 (2)	0.014 (2)	0.048 (3)
C18	0.0365 (15)	0.0428 (14)	0.0404 (14)	−0.0013 (12)	0.0012 (12)	0.0081 (12)
C19	0.069 (2)	0.0547 (18)	0.0415 (16)	0.0133 (17)	0.0076 (15)	−0.0144 (13)
S1	0.0219 (3)	0.0225 (3)	0.0302 (3)	0.0083 (2)	−0.0031 (2)	0.0019 (2)
O4	0.0245 (9)	0.0312 (8)	0.0486 (10)	0.0121 (7)	−0.0063 (8)	0.0022 (7)
O5	0.0330 (9)	0.0345 (9)	0.0417 (10)	0.0086 (8)	−0.0085 (8)	−0.0093 (7)
O6	0.0366 (10)	0.0325 (9)	0.0468 (10)	0.0173 (8)	0.0103 (8)	0.0150 (7)
C20	0.0357 (14)	0.0319 (12)	0.0333 (13)	0.0051 (11)	0.0015 (11)	0.0034 (10)

### Geometric parameters (Å, º)

**Table:**

O1—C10	1.365 (3)	C10—C11	1.410 (3)
O1—C14	1.431 (3)	C11—C12	1.451 (4)
O2—C8	1.364 (3)	C12—C13	1.323 (4)
O2—C18	1.420 (3)	C12—H12	0.9400
O3—C9	1.371 (3)	C13—C14	1.481 (4)
O3—C19	1.423 (4)	C13—H13	0.9400
N1—C1	1.341 (3)	C14—C15	1.441 (4)
N1—C2	1.362 (3)	C14—H14	0.9900
N1—H1N	0.85 (3)	C15—C17	1.458 (5)
N2—C2	1.330 (3)	C15—C16	1.463 (5)
N2—C3	1.345 (3)	C15—H15	1.007 (18)
N3—C2	1.325 (3)	C16—C17	1.500 (6)
N3—H3A	0.857 (17)	C16—H16A	0.965 (19)
N3—H3B	0.869 (17)	C16—H16B	0.988 (19)
N4—C3	1.322 (3)	C17—H17A	0.958 (19)
N4—H4A	0.871 (17)	C17—H17B	0.971 (19)
N4—H4B	0.863 (17)	C18—H18A	0.9700
C1—C4	1.347 (3)	C18—H18B	0.9700
C1—H1	0.9400	C18—H18C	0.9700
C3—C4	1.444 (3)	C19—H19A	0.9700
C4—C5	1.510 (3)	C19—H19B	0.9700
C5—C6	1.510 (3)	C19—H19C	0.9700
C5—H5A	0.9800	S1—O6	1.4442 (16)
C5—H5B	0.9800	S1—O5	1.4588 (17)
C6—C7	1.394 (3)	S1—O4	1.4658 (17)
C6—C11	1.395 (3)	S1—C20	1.763 (2)
C7—C8	1.390 (3)	C20—H20A	0.9700
C7—H7	0.9400	C20—H20B	0.9700
C8—C9	1.400 (3)	C20—H20C	0.9700
C9—C10	1.375 (3)		
			
C10—O1—C14	119.5 (2)	C12—C13—C14	122.0 (3)
C8—O2—C18	117.48 (19)	C12—C13—H13	119.0
C9—O3—C19	115.4 (2)	C14—C13—H13	119.0
C1—N1—C2	119.79 (18)	O1—C14—C15	109.1 (2)
C1—N1—H1N	121.2 (18)	O1—C14—C13	112.5 (2)
C2—N1—H1N	118.9 (18)	C15—C14—C13	118.4 (3)
C2—N2—C3	118.52 (18)	O1—C14—H14	105.2
C2—N3—H3A	119.3 (18)	C15—C14—H14	105.2
C2—N3—H3B	121.4 (19)	C13—C14—H14	105.2
H3A—N3—H3B	119 (3)	C14—C15—C17	123.6 (4)
C3—N4—H4A	118.4 (19)	C14—C15—C16	124.3 (3)
C3—N4—H4B	119.0 (18)	C17—C15—C16	61.8 (3)
H4A—N4—H4B	122 (3)	C14—C15—H15	110 (2)
N1—C1—C4	122.8 (2)	C17—C15—H15	108 (2)
N1—C1—H1	118.6	C16—C15—H15	120 (2)
C4—C1—H1	118.6	C15—C16—C17	58.9 (3)
N3—C2—N2	120.8 (2)	C15—C16—H16A	124 (3)
N3—C2—N1	117.59 (19)	C17—C16—H16A	112 (3)
N2—C2—N1	121.6 (2)	C15—C16—H16B	109 (3)
N4—C3—N2	117.50 (19)	C17—C16—H16B	118 (3)
N4—C3—C4	120.4 (2)	H16A—C16—H16B	120 (4)
N2—C3—C4	122.07 (19)	C15—C17—C16	59.3 (3)
C1—C4—C3	114.73 (19)	C15—C17—H17A	120 (3)
C1—C4—C5	123.42 (19)	C16—C17—H17A	120 (3)
C3—C4—C5	121.84 (18)	C15—C17—H17B	110 (3)
C6—C5—C4	114.54 (18)	C16—C17—H17B	120 (3)
C6—C5—H5A	108.6	H17A—C17—H17B	114 (4)
C4—C5—H5A	108.6	O2—C18—H18A	109.5
C6—C5—H5B	108.6	O2—C18—H18B	109.5
C4—C5—H5B	108.6	H18A—C18—H18B	109.5
H5A—C5—H5B	107.6	O2—C18—H18C	109.5
C7—C6—C11	119.7 (2)	H18A—C18—H18C	109.5
C7—C6—C5	119.6 (2)	H18B—C18—H18C	109.5
C11—C6—C5	120.8 (2)	O3—C19—H19A	109.5
C8—C7—C6	121.3 (2)	O3—C19—H19B	109.5
C8—C7—H7	119.3	H19A—C19—H19B	109.5
C6—C7—H7	119.3	O3—C19—H19C	109.5
O2—C8—C7	125.0 (2)	H19A—C19—H19C	109.5
O2—C8—C9	115.5 (2)	H19B—C19—H19C	109.5
C7—C8—C9	119.5 (2)	O6—S1—O5	113.41 (11)
O3—C9—C10	121.8 (2)	O6—S1—O4	113.82 (11)
O3—C9—C8	119.0 (2)	O5—S1—O4	111.29 (10)
C10—C9—C8	119.0 (2)	O6—S1—C20	105.55 (11)
O1—C10—C9	116.1 (2)	O5—S1—C20	105.63 (12)
O1—C10—C11	121.4 (2)	O4—S1—C20	106.36 (11)
C9—C10—C11	122.4 (2)	S1—C20—H20A	109.5
C6—C11—C10	118.2 (2)	S1—C20—H20B	109.5
C6—C11—C12	125.0 (2)	H20A—C20—H20B	109.5
C10—C11—C12	116.7 (2)	S1—C20—H20C	109.5
C13—C12—C11	120.6 (3)	H20A—C20—H20C	109.5
C13—C12—H12	119.7	H20B—C20—H20C	109.5
C11—C12—H12	119.7		
			
C2—N1—C1—C4	3.6 (3)	C7—C8—C9—C10	−0.3 (3)
C3—N2—C2—N3	−179.3 (2)	C14—O1—C10—C9	161.4 (2)
C3—N2—C2—N1	0.5 (3)	C14—O1—C10—C11	−23.3 (4)
C1—N1—C2—N3	174.6 (2)	O3—C9—C10—O1	0.6 (3)
C1—N1—C2—N2	−5.3 (3)	C8—C9—C10—O1	174.7 (2)
C2—N2—C3—N4	−174.6 (2)	O3—C9—C10—C11	−174.6 (2)
C2—N2—C3—C4	5.9 (3)	C8—C9—C10—C11	−0.5 (4)
N1—C1—C4—C3	2.4 (3)	C7—C6—C11—C10	−1.3 (3)
N1—C1—C4—C5	−178.4 (2)	C5—C6—C11—C10	178.4 (2)
N4—C3—C4—C1	173.2 (2)	C7—C6—C11—C12	−176.3 (2)
N2—C3—C4—C1	−7.3 (3)	C5—C6—C11—C12	3.4 (4)
N4—C3—C4—C5	−6.0 (3)	O1—C10—C11—C6	−173.7 (2)
N2—C3—C4—C5	173.5 (2)	C9—C10—C11—C6	1.3 (4)
C1—C4—C5—C6	20.0 (3)	O1—C10—C11—C12	1.8 (4)
C3—C4—C5—C6	−160.8 (2)	C9—C10—C11—C12	176.7 (2)
C4—C5—C6—C7	−96.5 (3)	C6—C11—C12—C13	−176.0 (3)
C4—C5—C6—C11	83.8 (3)	C10—C11—C12—C13	8.9 (4)
C11—C6—C7—C8	0.5 (3)	C11—C12—C13—C14	1.7 (5)
C5—C6—C7—C8	−179.1 (2)	C10—O1—C14—C15	165.4 (3)
C18—O2—C8—C7	−12.6 (3)	C10—O1—C14—C13	31.8 (4)
C18—O2—C8—C9	167.6 (2)	C12—C13—C14—O1	−21.4 (4)
C6—C7—C8—O2	−179.5 (2)	C12—C13—C14—C15	−150.4 (3)
C6—C7—C8—C9	0.3 (3)	O1—C14—C15—C17	149.3 (4)
C19—O3—C9—C10	−70.3 (3)	C13—C14—C15—C17	−80.3 (5)
C19—O3—C9—C8	115.5 (3)	O1—C14—C15—C16	72.7 (5)
O2—C8—C9—O3	−6.2 (3)	C13—C14—C15—C16	−156.8 (4)
C7—C8—C9—O3	174.0 (2)	C14—C15—C16—C17	113.3 (4)
O2—C8—C9—C10	179.5 (2)	C14—C15—C17—C16	−114.3 (4)

### Hydrogen-bond geometry (Å, º)

**Table:**

*D*—H···*A*	*D*—H	H···*A*	*D*···*A*	*D*—H···*A*
N1—H1*N*···O4^i^	0.85 (3)	1.99 (3)	2.827 (2)	168 (3)
N3—H3*A*···O5^i^	0.86 (2)	2.06 (2)	2.896 (3)	166 (2)
N3—H3*B*···O5^ii^	0.87 (2)	2.15 (2)	2.871 (3)	140 (2)
N4—H4*A*···N2^ii^	0.87 (2)	2.27 (2)	3.107 (3)	163 (2)
N4—H4*B*···O6	0.86 (2)	2.11 (2)	2.948 (3)	163 (2)

Symmetry codes: (i) *x*−1, *y*+1, *z*; (ii) −*x*, −*y*+1, −*z*.
